# Knowledge, Attitude, and Practices of White Spot Lesion Management Among Dental Professionals in South India: A Cross-Sectional Study

**DOI:** 10.7759/cureus.74877

**Published:** 2024-11-30

**Authors:** Revathy Parthasarathy, Srividhya Srinivasan, Sankar Vishwanath, Janani Karunakaran, Sangita Ilango, Nikesh Sakthi

**Affiliations:** 1 Conservative Dentistry and Endodontics, Sree Balaji Dental College and Hospital, Chennai, IND; 2 Conservative Dentistry and Endodontics, Sri Venkateswara Dental College and Hospital, Chennai, IND; 3 Conservative Dentistry and Endodontics, KSR Institute of Dental Science and Research, Tiruchengode, IND; 4 Conservative Dentistry and Endodontics, Chettinad Dental College and Research Institute, Chennai, IND; 5 Conservative Dentistry and Endodontics, Tagore Dental College and Hospital, Chennai, IND

**Keywords:** demineralization, fluoride, icdas, treatment choices, white spot lesion

## Abstract

Introduction

White spot lesions (WSLs) are opacities formed due to decalcification occurring in the enamel’s subsurface layer. These lesions are most commonly seen in patients during and after fixed orthodontic treatment due to undisturbed accumulation of plaque. Other factors that can predispose to WSLs are enamel hypomineralization, hypomaturation, hypoplasia, and disruption in the levels of salivary calcium, phosphate, bicarbonate, and fluoride. Though these lesions produce aesthetic mishaps, they go unattended because of the lack of clinical knowledge in interpretation and diagnosis.

Aim

This study aimed to determine the knowledge, attitude, and practice of dental professionals regarding the management of WSLs.

Materials and methods

Participants in a cross-sectional questionnaire-based survey included undergraduate practitioners, postgraduates, and specialists. Formulated in English, the questionnaire comprised 21 structured questions circulated through online media across India.

Results

The assessment of knowledge and awareness highlighted participants’ understanding of WSL, underscoring fundamental knowledge shared across dental professionals. The attitudes toward WSL treatment showed a similar belief among participants, including undergraduate practitioners and specialists, regarding the necessity of treating WSL. Furthermore, the analysis of practice patterns revealed consistent diagnostic methods among dental professionals, with specialists showing slightly higher adherence to drying techniques for enhanced visibility.

Statistical analysis

Data analysis was conducted using IBM SPSS Statistics software, version 23 (IBM Corp., Armonk, NY), with descriptive statistics presented in frequencies, percentages, and means. Comparisons were made across different categories of dental professionals to examine variations in responses and practices.

Conclusion

Because untreated WSL can progress into cavities, a thorough understanding of the etiology, clinical picture, and early diagnosis is crucial in planning the treatment and managing patients’ aesthetic demands and the tooth’s function.

## Introduction

Modern advancements in the field of dentistry have paved the way for conservative management and early detection of dental caries. Incipient caries are lesions that appear on the tooth surface as early signs of enamel demineralization. These incipient lesions are otherwise known as white spot lesions (WSLs) due to their chalky appearance on the tooth surface. White spot lesions indicate subsurface enamel demineralization on the smooth surface of the tooth. They can be classified as carious and non-carious, where carious lesions occur due to the demineralization of teeth and non-carious lesions occur due to developmental defects of enamel and fluorosis. Due to the dynamic demineralization-remineralization process, the integrity of the oral cavity and teeth is maintained [1]. Any change in this process leads to the formation of an initial carious lesion that eventually progresses into dental caries if left untreated. White spot lesions histologically have two surfaces: the superficial surface layer and the sub-surface or body of the lesion layer. The superficial layer is remineralized due to the constant flow of salivary remineralizing proteins, whereas the underlying subsurface layer remains demineralized because the macromolecular salivary proteins do not penetrate the subsurface layer of the enamel [2]. Due to the continuous diffusion of acids, decalcification occurs in the enamel’s subsurface layer, which in turn leads to cavitation in the enamel. The initiation of any carious lesion depends upon diet, host factors, microorganisms, and time. In WSLs, fixed orthodontic appliances serve as a major etiological factor because they allow undisturbed accumulation of plaque and prevent easy removal [3].

According to previous studies, WSLs occur in about 23% to 95% of the population, with men having a higher prevalence than women [4]. Patients with fixed orthodontic appliances have an increased risk of WSLs in the first weeks of treatment [5, 6], which increases to 40% within the first six months [6]. Treatment for WSLs ranges from non-invasive to minimally invasive treatment methods, including oral hygiene instructions; diet counselling; fluoride applications in the form of gels, toothpaste, varnishes, and foams; remineralizing and bleaching agents; lasers; and resin infiltration. If WSLs are not treated, these areas of demineralization lead to plaque accumulation, discolouration, and aesthetic mishaps and might eventually lead to cavitation [7]. Hence, the present study was formulated to evaluate the knowledge regarding the management of WSLs in clinical practice among various dental professionals in the South Indian population.

## Materials and methods

Dental professionals who are general practitioners, postgraduates, and academicians of various dental specialties were included in this study. Undergraduate students were excluded. An ethical clearance was obtained from the institutional human ethics committee of Chettinad Academy of Research and Education (CARE IHEC-II) before initiating the study (approval number: IHEC-II/0287/22).

A survey questionnaire was fabricated in the English language by Google Forms (Google Inc., Mountain View, CA) after estimating the sample size of about 246 by G*Power statistical software (Ver. 3.1 Heinrich-Heine-Universität Düsseldorf, Düsseldorf, Germany). The questions were initially validated by five clinical experts with at least 15 years of clinical training and professors from various dental colleges. The Google Forms link was circulated online after the final evaluation. The questionnaire contained details regarding the study motivation and an informed consent for participants to sign in advance (Appendix A). Demographic details such as age, years of experience, and qualifications were obtained, and the confidentiality of collected data was maintained.

The survey questions included an evaluation of oral health practices, dental knowledge, and the attitude of the dentist toward treating WSLs in a sequential manner. The questions were framed in a multiple-choice pattern, and space was also provided for participants to write their responses. The questionnaire included knowledge-based questions that could be answered either yes or no. Attitude-based questions revealed the dentist’s perception in analyzing WSLs. The questionnaire also focused on the percentage of WSL cases that the dentist had treated successfully with the aim of assessing the clinician’s practical knowledge in diagnosing the lesion and their clinical proficiency. In the determined sample size, 246 responses were obtained over a two-month period.

The sociodemographic profile and information regarding knowledge, attitude, and practice of managing WSLs among various dental professionals collected using Google Forms were later transferred to Microsoft Excel (Microsoft Corp., Redmond, WA). Data analysis was conducted using IBM SPSS Statistics software, version 23 (IBM Corp., Armonk, NY), with descriptive statistics presented in frequencies, percentages, and means. Comparisons were made across different categories of dental professionals to examine variations in responses and practices.

## Results

**Figure 1:**
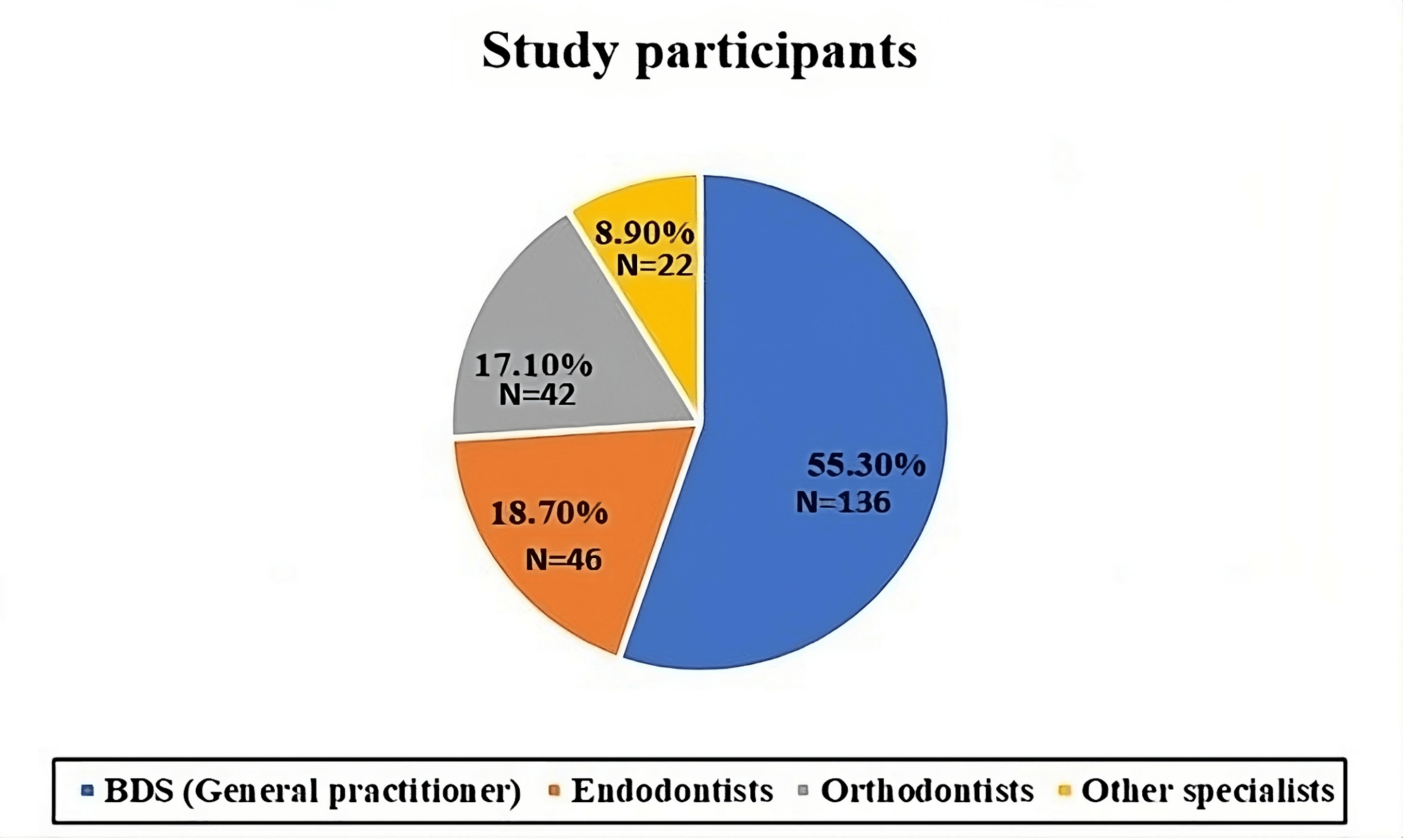
Distribution of various dental professionals in the study BDS: Bachelor of Dental Surgery

The current study involved investigating the knowledge, attitudes, and practices regarding WSL among 246 dental professionals, including Bachelor of Dental Surgery (BDS) general practitioners and specialists, including Master of Dental Surgery (MDS) practitioners such as endodontists, orthodontists, and postgraduates. The demographic analysis appears in Figure 1. The specialists had clinical experience ranging from 0 to two years. Furthermore, they showed higher mean ages when compared to general practitioners.

The assessment of knowledge and 100% awareness of WSLs highlighted an understanding among all participants, underscoring fundamental knowledge shared across dental professionals (Table 1). These findings reflect the specialists’ comprehensive understanding and specialized training in addressing early signs of dental caries.

**Table 1:** Knowledge and awareness assessment of dental professionals regarding white spot lesions (WSLs) and caries detection techniques (N=246) Notably, specialists, particularly endodontists, showed higher familiarity with advanced concepts such as the International Caries Detection and Assessment System (ICDAS) and the combined use of fluoride and chlorhexidine for managing WSL. Additionally, specialists exhibited a greater consensus on the urgency of treating WSLs post orthodontic treatment and the effectiveness of xylitol gums in WSL management compared to general practitioners. MDS: Master of Dental Surgery

Questions		General practitioners n (%)	Specialists (including MDS practitioners and postgraduates) n (%)		
			Endodontists	Orthodontists	Other specialists
Q1	Are you aware of white spot lesions (WSLs)?				
	Yes	136 (100%)	46 (100%)	42 (100%)	22 (100%)
	No	0	0	0	0
Q2	Are you aware of the International Caries Detection and Assessment System (ICDAS)?				
	Yes	90 (66.2%)	38 (82.6%)	25 (59.5%)	13 (59.1%)
	No	46 (33.8%)	8 (17.4%)	17 (40.5%)	9 (40.9%)
Q3	Does combined use of fluoride and chlorhexidine (CHX), produce effect on WSLs?				
	Yes	32 (23.5%)	25 (54.3%)	14 (33.3%)	9 (40.9%)
	No	18 (13.2%)	8 (17.4%)	6 (14.3%)	2 (9.1%)
	Not aware of it	86 (63.2%)	13 (28.3%)	22 (52.4%)	11 (50%)
Q4	Do post-orthodontic WSLs require immediate treatment?				
	Yes	62 (45.6%)	29 (63%)	24 (57.1%)	13 (59.1%)
	No	34 (25%)	9 (19.6%)	7 (16.7%)	3 (13.6%)
	Not aware of it	40 (29.4%)	8 (17.4%)	11 (26.2%)	6 (27.3%)
Q5	Does xylitol gums help in treating WSLs?				
	Yes	42 (30.9%)	23 (50%)	13 (31%)	7 (31.8%)
	No	38 (27.9%)	8 (17.4%)	9 (21.4%)	4 (18.2%)
	Not aware of it	56 (41.2%)	15 (32.6%)	20 (47.6%)	11 (50%)
Q6	The first sign of demineralization as WSL can form within				
	Correct answer (4 weeks)	41 (30.1%)	15 (32.6%)	14 (33.3%)	6 (27.3%)
Q7	Frequency of application of fluoride varnish for permanent teeth				
	Correct answer (2-4 times/year)	110 (80.9%)	37 (80.4%)	36 (85.7%)	19 (86.4%)
Q8	The most common method of micro-abrasion uses				
	Correct answer (18% Hydrochloric acid)	31 (22.8%)	16 (34.8%)	5 (11.9%)	6 (27.3%)
Q9	Refractive index of enamel (RI)				
	Correct answer (1.62 – 1.65)	105 (77.2%)	38 (82.6%)	19 (45.2%)	18 (81.8%)
Q10	Resin infiltration works on the basic principle of				
	Correct answer (occlusion of pores that provides diffusion of acids)	49 (36%)	16 (34.8%)	22 (52.4%)	9 (40.9%)

The attitudes toward WSL treatment (Table 2) showed a similar belief among participants, including general practitioners and specialists, regarding the necessity of treating WSLs.

**Table 2:** Dental professionals' attitude towards the treatment of white spot lesions (WSLs) (N=246) MDS: Master of Dental Surgery

Question		General practitioners n (%)	Specialists (including MDS practitioners and postgraduates) n (%)		
			Endodontists	Orthodontists	Other specialists
Q1	Is it necessary to treat WSLs?				
	Yes	120 (88.2%)	39 (84.8%)	32 (76.2%)	18 (81.8%)
	No	16 (11.8%)	7 (15.2%)	10 (23.8%)	4 (18.2%)

Furthermore, the analysis of practice patterns (Table 3) revealed consistent diagnostic methods among the various dental professionals. 

**Table 3:** Dental professionals' practice patterns and preferences in the management of white spot lesions (WSLs) (N=246) Consistent diagnostic methods among dental professionals, with specialists showing slightly higher adherence to drying techniques for enhanced visibility was noted. While the majority of the general practitioners and specialists opted for conservative treatment approaches for WSL, a considerable proportion of specialists showed a higher preference towards micro-invasive techniques like resin infiltration and remineralizing agents to preserve tooth structure and achieve aesthetic outcomes. This emphasizes a shift towards non-invasive and minimally invasive interventions among specialists. Lastly, among the dental professionals, specialists showed varying success rates; particularly, endodontists reported higher success rates when compared to orthodontists and general practitioners in non-invasive treatment for WSL. MDS: Master of Dental Surgery

Questions		General practitioners n (%)	Specialists (including MDS practitioners and postgraduates) n (%)		
			Endodontists	Orthodontists	Other specialists
Q1	How do you diagnose a WSL?				
	Correct answer (By drying the tooth)	97 (71.3%)	36 (78.3%)	34 (81%)	17 (77.3%)
Q2	Topical fluoride regimen, that you would recommend for treating deeper parts of the lesion and for better esthetic outcome				
	Correct answer (Lower concentration of fluoride initially)	77 (56.6%)	22 (47.8%)	22 (52.4%)	10 (45.5%)
Q3	What would be your treatment of choice to treat a WSL?				
	Non-invasive treatment (conservative approach)	93 (68.4%)	30 (65.2%)	18 (42.9%)	14 (63.6%)
	Invasive treatment (restoration, tooth bleaching, micro-abrasion)	21 (15.4%)	4 (8.7%)	6 (14.3%)	2 (9.1%)
	Micro-invasive technique (Resin infiltration)	22 (16.2%)	12 (26.1%)	18 (42.9%)	6 (27.3%)
Q4	In case of conservative management of WSL, what is your preference?				
	Oral hygiene instructions	15 (11%)	6 (13%)	1 (2.4%)	0
	Fluoride mouth-rinse/ toothpaste	26 (19.1%)	5 (10.9%)	7 (16.7%)	5 (22.7%)
	Fluoride gel/ varnish application	43 (31.6%)	11 (23.9%)	12 (28.6%)	7 (31.8%)
	Re-mineralizing agents	52 (38.2%)	24 (52.2%)	22 (52.4%)	10 (45.5%)
Q5	In case of invasive approach for WSL, what is your preference?				
	Composite restoration	36 (26.5%)	21 (45.7%)	14 (33.3%)	7 (31.8%)
	Tooth bleaching	23 (16.9%)	2 (4.3%)	6 (14.3%)	2 (9.1%)
	Veneers	27 (19.9%)	4 (8.7%)	11 (26.2%)	6 (27.3%)
	Micro-abrasion	50 (36.8%)	19 (41.3%)	11 (26.2%)	7 (31.8%)
Q6	The percentage of WSL cases that you have diagnosed and treated				
	Less than 10%	86 (63.2%)	36 (78.3%)	25 (59.5%)	18 (81.8%)
	10 -30%	23 (16.9%)	6 (13%)	12 (28.6%)	1 (4.5%)
	30-50%	17 (12.5%)	1 (2.2%)	3 (7.1%)	2 (9.1%)
	More than 50 %	10 (7.4%)	3 (6.5%)	2 (4.8%)	1 (4.5%)

## Discussion

The current study is one among few studies that was aimed at focusing on the diagnosis and management of WSL. To the best of our knowledge, the present study is the first to compare general dental practitioners, postgraduates, and academicians of various specialties, emphasizing their clinical management of WSLs, whereas previous questionnaire studies had only focused on orthodontists or dental students alone [1, 8-10].

Previous researchers have stated that WSLs occur most commonly in patients with a fixed orthodontic appliance, with the incidence and prevalence percentages ranging from 23.4% to 75.6% and 33.8% to 97%, respectively [11,12]. They also indicated that in the Indian population, more than 75% of patients with a fixed orthodontic appliance had a higher prevalence of WSL [11].

In the current study, general practitioners accounted for the majority of participants (55.3%), whereas specialists together comprised 53% but had diverse expertise, with significant numbers of endodontists and orthodontists because of their preventive and aesthetic treatment of WSLs.

The International Caries Detection & Assessment System (ICDAS), which permits standardized caries detection and assessment of various stages of dental caries, was developed in 2002 and later revised in 2009. The International Caries Classification and Management System was introduced later as a radiographic and clinical assessment method to categorize carious lesions [13]. In the current study regarding a particular question about awareness of ICDAS, the majority of general practitioners (66.2%) and a higher proportion of specialists, particularly endodontists (82.6%), showed greater familiarity with the ICDAS system. Orthodontists and other specialists also exhibited notable awareness levels (59.5% and 59.1%, respectively) regarding ICDAS.

In this study, when questioned about the necessity to treat WSL, most respondents, including general practitioners and specialists, answered that such treatment is essential. According to the ICDAS scoring, when a lesion is visible after drying the teeth, it is classified as Code 1, and if it is visible under both wet and dry conditions, it is classified as Code 2. Based on this, if a non-cavitated caries is inactive, it does not require any treatment. Non-cavitated approximal lesions require resin infiltration alone or resin infiltration in combination with 5% sodium fluoride (NaF) varnish for three to six months [14]. The protocol for the management of non-cavitated lesions occurring on the labial and palatal smooth surfaces advises the use of 1.23% acidulated phosphate fluoride or 5% NaF for three to six months [15].

When assessing the combined use of fluoride and chlorhexidine (CHX) in managing WSLs, awareness levels varied among specialist subgroups. Endodontists (54.3%), orthodontists (33.3%), and other specialists (40.9%) demonstrated relatively higher awareness compared to general practitioners (23.5%). However, a significant proportion of respondents across all groups (ranging from 28.3% to 63.2%) showed a lack of awareness regarding this therapeutic approach. Among the dental varnishes available, fluoride varnish has proven to be safe and effective against the development of new carious lesions [16,17]. Apart from fluoride, CHX as varnish performs better due to the higher concentration of chlorhexidine and better contact time. The combined use of CHX and fluoride seems to have a synergistic effect, as CHX reduces the acid production by its antimicrobial action and fluoride works on the remineralization of the white spot lesion [18]. Chlorhexidine mouthwash (0.12%) can also be used along with fluoride therapy [18,19].

Another agent that has proven to be synergistic with the above-mentioned combination is xylitol, a non-fermentable sugar [20]. Xylitol-incorporated chewing gums have proven to increase the production of stimulated saliva, which has a higher concentration of bicarbonate and phosphate. This improves the buffering action of the saliva, thus preventing demineralization. Along with this calcium, phosphate, and hydroxyl ions are also produced in higher concentrations, which are accountable for remineralization [21]. In the current study, when questioned about the effectiveness of xylitol gums in treating WSLs, a considerable proportion of participants (ranging from 32.6% to 50%) reported being unaware of the therapeutic role of xylitol gums.

In questions based on practice patterns and preferences in the management of WSLs, when recommending a topical fluoride regimen to treat deeper parts of the lesion and improve aesthetic outcomes, general practitioners (56.6%) favoured initiating treatment with a lower fluoride concentration. In contrast, specialists’ preferences varied, with only 47.8% of endodontists, 52.4% of orthodontists, and 45.5% of other specialists opting for lower fluoride concentrations. Though higher concentrations of fluoride are chosen in clinical practice, there are increased chances of mineralization occurring only on the surface layer, thereby acting as a hindrance to calcium and phosphate ions penetrating into the deeper layers. This process is known as lamination. Thus, when low concentrations of fluoride are applied to a WSL, slow remineralization occurs from its deeper surface to its superficial layer, thereby producing aesthetic results [22].

Regarding treatment choice for WSLs, a significant proportion of dental professionals across all categories showed a preference for non-invasive or conservative approaches. Mild post-orthodontic WSLs have the property to remineralize naturally over a period of time within the first six months [19,23]. Hence remineralizing agents and fluoride therapy can be advised. Bleaching can be a treatment of choice to camouflage the affected area from the normal site [24]. In cases where bleaching did not work, micro-abrasion followed by casein phosphopeptides-amorphous calcium phosphate (CPP-ACP) therapy has proven to produce effective results [25].

Conversely, for cases requiring invasive interventions, preferences diverged across general practitioners and specialists. Orthodontists and endodontists exhibited a higher preference towards composite restorations, micro-abrasion, and interest in using veneers for managing WSLs, highlighting the approach towards achieving aesthetic outcomes through invasive treatment modalities. Apart from these techniques, resin infiltration has produced evident results in masking the WSL. It is a micro-invasive technique that occludes the micro-porosities of the enamel with low-viscosity light-curing resins. It is the preferred treatment option for smooth surface caries occurring on the facial and proximal surfaces, producing esthetic results [26].

Though the incidence of WSLs appears to be higher among various dental professionals, the percentage of cases diagnosed and treated seems to be low. This might be due to a lack of resources, patient awareness, limited access to advanced diagnostic tools, and cost. The application of theoretical knowledge in the preventive management of such lesions also seems to be low. As the management of WSLs needs a multi-factorial approach, further importance should be given to the early diagnosis and minimally invasive inter-disciplinary management of such lesions.

The limitations of the current study are that there was a low response rate and selection bias from online survey distribution. As the study focuses on a particular population, this has affected the generalizability of the result. 

## Conclusions

It is essential that all dental practitioners formulate a caries risk assessment chart for patients with active and non-active caries lesions to determine the occurrence of new caries and re-emission of old ones. Though preventive treatment options have been available for more than a decade, the awareness about incorporating such preventive methods in clinical practice still seems to be scarce. Hence, practitioners should be aware of proper evaluation, assessment, treatment planning, and recent advancements in the preventive management of initial caries lesions. Also, the aforementioned must be taught to students in their undergraduate curriculum with the aim of showing them how to decrease the incidence of cavitation efficiently.

## Appendices

Appendix A

The Study Questionnaire

**Table 4:** Questionnaire distributed to the study group

S.No	Question	Options
DEMOGRAPHIC DATA		
1.	Your highest qualification?	a) BDS general practitioner
		b) MDS practitioner
		c) Postgraduate - Endo
		d) Postgraduate Others - specify _________
2.	If you are an MDS practitioner, specify your specialty	_______________
3.	Age	_______________
4.	Years of clinical experience	a) 0-2 years
		b) 2-5 years
		c) 5-10 years
		d) >10 years
KNOWLEDGE-BASED QUESTIONS		
1.	Are you aware of white spot lesions (WSL)?	a) Yes
		b) No
2.	Are you aware of the International Caries Detection & Assessment System (ICDAS)?	a) Yes
		b) No
3.	Does the combined use of fluoride and chlorhexidine (CHX) produce an effect on WSL?	a) Yes
		b) No
		c) Not aware of it
4.	Does post-orthodontic WSL require immediate treatment?	a) Yes
		b) No
		c) Not aware of it
5.	Does xylitol gum help in treating WSL?	a) Yes
		b) No
		c) Not aware of it
6.	The first sign of demineralization as WSL can form within	a) 2 weeks
		b) 3 weeks
		c) 4 weeks
7.	The most common method of micro-abrasion uses	a) 18% Hydrochloric acid
		b) 37% Phosphoric acid
		c) 15% Hydrochloric acid
8.	Refractive index of enamel (RI)	a) 1.62 - 1.65
		b) 2.32 - 2.33
		c) 3.23 - 3.35
9.	Resin infiltration works on the basic principle of	a) Controlled removal of enamel surface
		b) Occlusion of pores that provides diffusion of acids
		c) Increasing the amount of calcium and phosphate ions
		d) Alteration in chemical composition and surface morphology
ATTITUDE-BASED QUESTIONS		
1.	Is it necessary to treat a white spot lesion?	a) Yes
		b) No
PRACTICE-BASED QUESTIONS		
1.	How do you diagnose a WSL?	a) By drying the tooth
		b) Without drying the tooth
		c) Radiography
		d) Transillumination
		e) Others - specify?
2.	Topical fluoride regimen you would recommend for treating deeper parts of the lesion and for a better aesthetic outcome	a) High concentration of fluoride throughout treatment
		b) Lower concentration of fluoride initially
		c) None of the above
3.	What would be your treatment of choice to treat a WSL?	a) Non-invasive treatment (conservative approach)
		b) Invasive treatment (restoration, tooth bleaching, micro-abrasion)
		c) Micro-invasive technique (Resin infiltration)
4.	For conservative management of WSL, what is your preference?	a) Oral hygiene instructions
		b) Fluoride mouth-rinse/toothpaste
		c) Fluoride gel/varnish application
		d) Re-mineralizing agents
5.	For an invasive approach, your treatment of choice	a) Composite restoration
		b) Tooth bleaching
		c) Veneers
		d) Micro-abrasion
6.	The percentage of WSL cases that you have diagnosed and treated	a) Less than 10%
		b) 10-30%
		c) 30-50%
		d) More than 50%
